# High expression of calreticulin indicates poor prognosis and modulates cell migration and invasion via activating Stat3 in nasopharyngeal carcinoma

**DOI:** 10.7150/jca.35362

**Published:** 2019-08-29

**Authors:** Yaqian Han, Qianjin Liao, Heran Wang, Shan Rao, Pin Yi, Lu Tang, Yutong Tian, Linda Oyang, Hui Wang, Yingrui Shi, Yujuan Zhou

**Affiliations:** 1 Hunan Cancer Hospital and The Affiliated Cancer Hospital of Xiangya School of Medicine, Central South University, 283 Tongzipo Road, Changsha 410013, Hunan, China.; 2 University of South China, Hengyang, 421001, Hunan, China.

**Keywords:** nasopharyngeal carcinoma, calreticulin, prognosis, progression, STAT3

## Abstract

**Objective**: Emerging evidence suggests that calreticulin (CALR) has great impacts on the tumor formation and progression of various cancers, but the role of CALR remains controversial. We investigated the expression and clinical significance of CALR in nasopharyngeal carcinoma (NPC).

**Methods**: Immunohistochemistry was used to detect the expression of CALR in NPC tissues, and the correlation of CALR with clinicopathological characteristics and prognosis were analyzed. The cell functions of CALR in NPC cells were also performed* in vitro*.

**Results**: Compared with non-tumor nasopharyngeal epithelium (NPE) tissues, CALR expression was markedly up-regulated in NPC tissues (P < 0.001), and the high expression of CALR was positively associated with advanced clinical stage (P=0.003) and metastasis (P=0.023). Compared to the patients with low expression of CALR, patients who displayed high expression of CALR may achieve a poorer progression-free survival (PFS) and overall survival (OS) (P < 0.001). Furthermore, multivariate analysis showed that high expression of CALR was an independent predictor of poor prognosis. In addition, we found that knockdown of CALR significantly inhibited the proliferation, migration and invasion of CNE2 and HONE1 cells *in vitro*, and the mechanism might be associated with inactivation of Stat3 signaling pathway.

**Conclusion**: CALR may promote NPC progression and metastasis via involving Stat3 signaling pathway, and can be regarded as an effective potential predictor for progression and prognosis of NPC.

## Introduction

Nasopharyngeal carcinoma (NPC) is one type of head and neck malignancies, and has a notable high prevalence in southern China^1^. Multiple studies found that NPC is closely associated with EB virus infection, environment, genetic susceptibility^2^-^4^. Several potential biomarkers have been correlated with the outcome^4^, ^5^. Nonetheless, these prognostic factors have limited utility in terms of predicting survival.

Calreticulin (CALR/CRT) protein is one of commonly expressed and highly conserved endoplasmic reticulum calcium protein^6^, which can regulate multiple physiological function through regulating calcium balance, assisting proper protein folding, participating the processes of apoptosis, adhesion, angiogenesis and autoimmune reactions^6^, ^7^. An increasing number of studies have demonstrated that CALR may be involved in the tumor initiation, progression, and metastasis, suggesting that CALR is a key therapeutic target in cancer^6^-^9^. CALR was highly expressed in gastric cancer, breast cancer, and other tumors, which was related to migration, invasion and abnormal apoptosis of tumor cells^10^-^12^. In gastric cancer, CALR can promote tumor angiogenesis, local invasion and lymph node metastasis, which is a good biomarker of prognosis in gastric cancer^13^. However, to date, the expression of CALR and associated clinical value are still unclear in NPC. In addition, our previous studies have indicated that CALR expression was down-regulated in response to LPLUNC1 (long palate, lung and nasal epithelium clone) overexpression, as detected using 2-D DIGE^14^, and our studies have indicated that LPLUNC acts as a potential tumor suppressor gene in NPC^15^-^18^. Thus, there may be a close relationship between CALR and NPC, and we speculated that the CALR might play an important role in the occurrence and development of NPC. To verify the speculation, we investigated the expression of CALR in the NPC tissues and evaluated whether CALR might play a critical role in NPC progression and prognosis as a potential prognostic biomarker.

## Materials and Methods

### Tumor samples

As our previous study^14^, NPC samples (256 cases) and non-tumor nasopharyngeal epithelium (NPE) (131 cases) were collected in Xiangya Hospital and the Second Xiangya Hospital (Changsha, PR China) during 2002 to 2004. All biopsies were immediately fixed in 4% buffered paraformaldehyde, routinely processed and embedded in paraffin, and then prepared the NPC tissue microarray. The clinicopathologic characteristics were listed in Table 1. Among all of the NPC patients, we followed up 81 patients to do survival analysis. The time of following up was from 4 to 95 month, and average was 57 month.

For the mRNA expression study, another 36 NPC tissues and 15 NPE samples were obtained from patients in the Hunan Cancer Hospital/The Affiliated Cancer Hospital of Xiangya School of Medicine (Changsha, China) in 2013. All tissue samples were quickly frozen in liquid nitrogen and stored at -80°C until laser-capture micro-dissection (LCM). We used a LEICA CM 1900 (Leica, Solms, Germany) for frozen sections and the Leica AS LMD system (Leica) to obtain the pure tissues.

All of the individuals participating in this project signed the informed consent form and their clinicopathologic characteristics, such as name, sex, age, metastasis and clinical stages were recorded.

### Cells and cell culture

NP69 cell line and all the NPC cell lines (CNE1, CNE2, HONE1, C666-1, HNE1, HNE2 and 5-8F) were obtained from the Cancer Research Institute of Central South University. The cells were cultured in RPMI-1640 (Gibco, Logan, UT, USA). The media were supplemented with 12% FBS (Zeta Life, France), 100 μg/ml penicillin, and 100 U/ml streptomycin (Gibco, Life Technologies, USA) at 37 °C in a 5% CO_2_ incubator.

### Immunohistochemistry (IHC)

Immunohistochemistry was done from paraffin-embedded tissue sections, and the CALR expression in individual NPC and NPE specimens was characterized by IHC using an immunohistochemistry kit (ComWin Biotech Co.,Ltd., Beijing, China) and following the manufacturer instructions. Slides were incubated with the primary antibody monoclonal rabbit anti- CALR (1:500, Abcam, Cambridge, UK) for overnight at 4ºC. Negative rabbit serum was used instead of the primary antibody as negative control. The specimens were visualized with 3, 3'-diaminobenzidine and counterstained with hematoxylin. The staining was brown or tan, and located in the cytoplasm or nucleus.

### Immunohistochemical evaluation

A semiquantitative scoring criterion for IHC was used in which the staining intensity and positive areas were recorded as reported previously^14^. Briefly, the intensity of anti-CALR staining was scored by 0-3, according to the standards of 0 (no staining), 1 (weak staining), 2 (medium staining), and 3 (strong staining). The percentage of reactivity was scored as follows: 0 (no positive tumor cells), 1 (<10% positive tumor cells), 2 (10-50% positive tumor cells) and 3 (>50% positive tumor cells). Individual samples were evaluated by at least two pathologists in a blinded manner, and those samples with inconsistent scores were further discussed and decided. For the statistical analysis, the score was obtained by multiplying the intensity and reactivity rates. Scores of < 4 suggested negative expression, and the remainder were classified as positive expression.

### Western blotting

As reported previously^15^, cells were lysed in RIPA buffer in the presence of Protease Inhibitor Cocktail and PhoSTOP (Roche, Basel, Switzerland). Protein was quantified using a BCA Protein Assay Kit (Pierce Biotechnology, Rockford, IL, USA). Protein (30μg) was separated using 10% sodium dodecyl sulfate polyacrylamide gel electrophoresis and transferred onto polyvinylidene fluoride membranes (PVDF) (Millipore, Billerica, MA, USA). The membranes were blocked with 5% non-fat milk in Tris-buffered saline and then incubated with primary antibodies at 4 °C overnight. Membranes were washed three times in TBST solution for 10 min each and then incubated with secondary antibodies. Signals were detected by an enhanced chemiluminescence detection system as the manufacturer's protocol (Bio-Rad, Hercules, CA, USA). The primary antibodies as follows: CALR (A1066#) rabbit polyclonal antibody was purchased from ABclonal Biotechnology Co., Ltd (Hubei, China), Stat3 (12640#) and p-Stat3 (9145#) rabbit monoclonal antibody was purchased from Cell Signaling Technology (Boston, USA), GAPDH (60004-1-Ig) mouse monoclonal antibody was purchased from Proteintech Company (Chicago, USA).

### RNA isolation and real-time PCR

Total RNA was isolated using Trizol (Invitrogen, CA) according to the manufacturer's instructions, and reversely transcribed into complementary DNA using AMV reverse transcriptase (Promega, San Luis Obispo, CA, USA). The levels of target gene mRNA transcripts were determined by qRT-PCR using specific primers and a SYBR-green-containing PCR kit (GenePharma, Shanghai, China). The sequences of primers were forward 5'-TCTCAGTTCCGGCAAGTTCT-3' and reverse 5'-TTCTGAGTCTCCGTGCATGT-3' for CALR (232 bp); forward 5'-GAAGGTGAAGGTCGGAGTC-3' and reverse 5'-GAAGATGGTGAT GGGATTTC-3' for GAPDH (226 bp). The relative levels of individual gene mRNA transcripts to control GAPDH were determined.

### Cck-8 assay

The Cck-8 assay was used to detect the proliferation rate of tumour cells. Cells of CNE2 and HONE1 were stably transfected with interfering plasmids and control vectors, respectively. Cells were trypsinized and seeded in 96-well plates at a density of 5×10^3^ cells/well in 200 μl of complete medium. Then, 10 μl of 5 mg/ml CCK-8 (Sigma, USA) was placed in each well at 0, 24, 48, and 72 h and incubated at 37 °C for 4 h. The absorbance was measured at 460 nm.

### Cell migration and invasion assays

A transwell chamber (8μm, 24-well format; Corning, USA), with or without a diluted Matrigel (BD Biosciences, New Jersey, USA) coating, was used to assess the migration and invasion of cultured cells. Briefly, 5×10^4^/200μl of cells (migration assays) and 1×10^5^/200μl of cells (invasion assays) were seeded in serum-free RPMI-1640 to the top chamber, the cells in the top chamber that had not migrated through the filter were wiped off with a cotton swab, while those that had migrated to the bottom surface were fixed in 4% paraformaldehyde for 30 min and stained with 0.1% crystal violet, then counted under a microscope. Migration and invasion rates are expressed as the ratio of the treated group value to the control group value.

### Wound-healing assay

Cells were seeded into 6-well plates (Corning, USA) in triplicate in adequate numbers for growth and attachment. The artificial “wound” was scratched by a 10μl pipette tip after cells were grown to 80% confluence, washed gently in PBS until there were no floating cells and then incubated in medium containing 3% FBS. Gap size was measured 0h, 24h, 48h, and the wound areas were then photographed using an inverted microscope.

### Statistical analysis

All analyses were performed using SPSS 15.0 program for Windows software package (SPSS, Chicago, IL, USA). Statistical significance between groups within experiments was determined by the One-way ANOVA and Student t-test. The chi-square test was used to determine whether two groups had distinct gene expression levels. Survival was estimated using the Kaplan-Meier method and compared by log-rank test. Multivariate logistic analyses using a stepwise Cox regression model after adjusting for baseline characteristics. A P-value of < 0.05 was considered statistically significant.

## Results

### CALR is overexpressed in NPC

**Figure 1:**
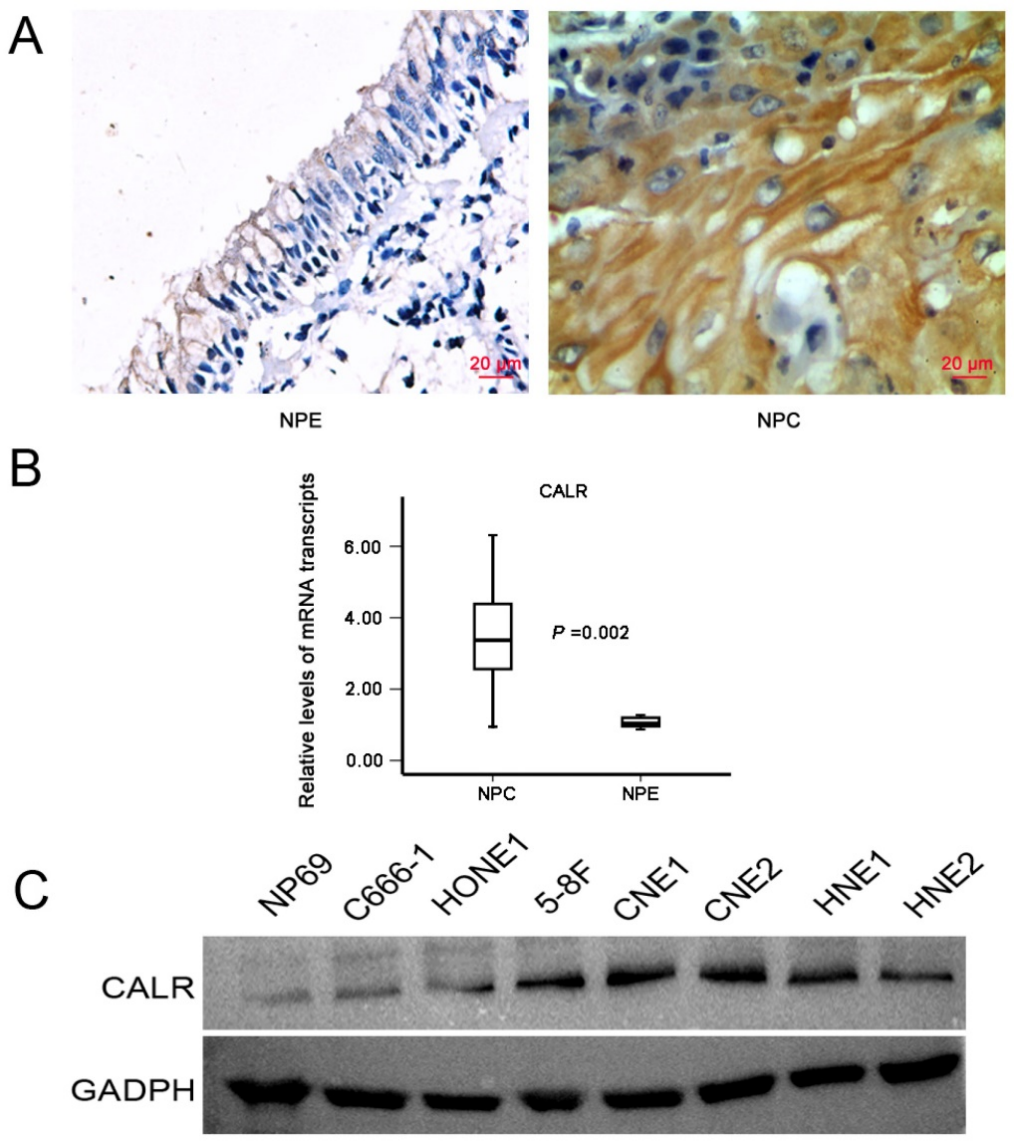
** Expression of CALR in NPC tissues and cells.** A: Representative IHC staining of **CALR** in NPC tissues (magnification×400, scale bars 20μm); B: qRT-PCR analysis of the relative expression levels of CALR in 36 NPC specimens and 15 NPE specimens; C: Detection the expression of CALR in NP69 cell line and NPC cell lines by western blotting. NPC: nasopharyngeal carcinoma, NPE: non-tumor nasopharyngeal epithelium.

Immunohistochemistry was used to measure the expression of CALR in NPC and NPE tissues. CALR was positively expressed mainly in the cytoplasm, showing obvious pale brown. Representative photographs for immunostaining were shown in Fig. 1A. Only a total of 16.8% (22/131) was high expression in NPE tissues, while a total of 64.1% (164/256) was high expression in NPC tissues(X^2^ =77.564, P < 0.001), suggesting CALR protein expression significantly increased in NPC tissues. Moreover, to further validate the level of CALR expression in the NPC tissues, we applied laser capture microdissection (LCM) to ensure the purity of NPC and NPE tissues, and detected the expression of CALR in mRNA level by qRT-PCR. Accord with the protein level, the mRNA expression of CALR was up-regulated significantly in NPC tissues (P < 0.001, Fig. 1B). Compared with normal human nasopharyngeal epithelium cell line (NP69), CALR was also overexpressed in four NPC cell lines (CNE1, CNE2, HONE1, C666-1, HNE1, HNE2 and 5-8F) (Fig. 1C). These results suggested that CALR was highly expressed in NPC, which related to the development and progression of NPC.

### Associations between CALR protein expression and clinicopathological parameters

Next, to clarify the clinical significance of the expression of CALR in NPC, the relationship between the expression of CALR and clinicopathological parameters was analyzed. As shown in Table 1, the increased CALR expression appeared to be associated with advanced clinical stage (P=0.003) and metastasis (P=0.023) of NPC, while not significant with gender (P =0.325) and age (P =0.123). Therefore, these results suggested that high expression of CALR was related to the progression of NPC, CALR could represent predictive factors of NPC.

### CALR high expression correlated with poor prognosis of NPC

**Figure 2:**
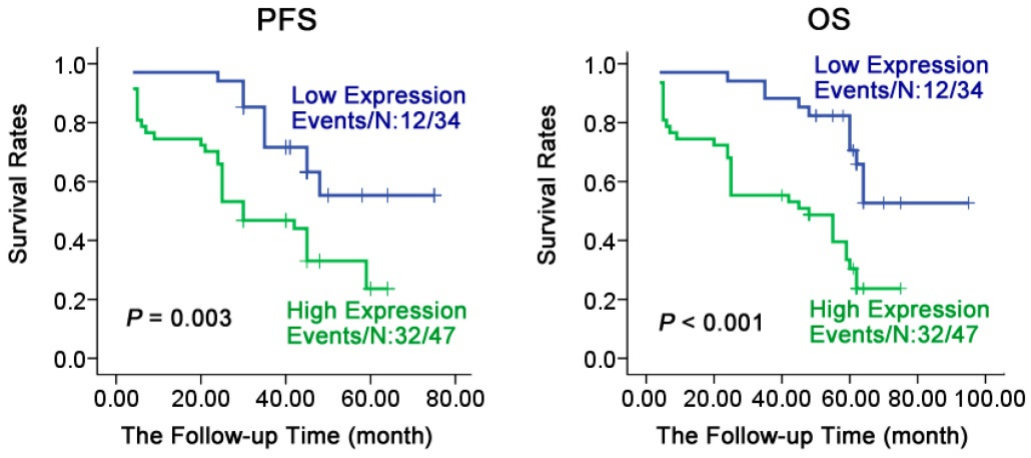
** Survival analysis of patients with varying levels of CALR expression in NPC.** Kaplan-Meier estimated progression-free survival (PFS) and overall survival (OS) for NPC patients according to the expression levels of CALR protein in 81 NPC patients. P values were obtained by using the log-rank test. N, The number of cases; Events, the number of cases, who had recurrent tumor or died during the follow-up period.

To understand the relationship of prognosis with expression of CALR in NPC, Kaplan-Meier method analysis the survival curve. As shown in Fig. 2, the expression of CALR was closely associated with the progression-free survival (PFS) and overall survival (OS) of NPC. Patients with NPC having a low expression of CALR had a significantly longer PFS and OS compared with the patients having high expression (61.023 ± 3.735 vs 33.758 ± 3.243, X^2^ = 14.901, *P* < 0.001; 77.111 ± 4.697 vs 39.785 ± 3.884, X^2^ =20.275, *P* < 0.001). Furthermore, multivariate regression analysis showed (Table 2) that CALR protein expression was significantly related to the PFS and OS, NPC patients who had high expression of CALR protein, had a shorter PFS (HR= 2.564, P = 0.013) and OS (HR= 3.179, P =0.003), while the age, gender, metastasis and clinical staging had no significant association with PFS or OS of patients through COX multi-factor regression. These findings suggested that the high expression of CALR was a potential molecular marker for poor prognostic monitoring in NPC.

### Knockdown of CALR inhibited cell proliferation, migration and invasion in NPC cells

**Figure 3:**
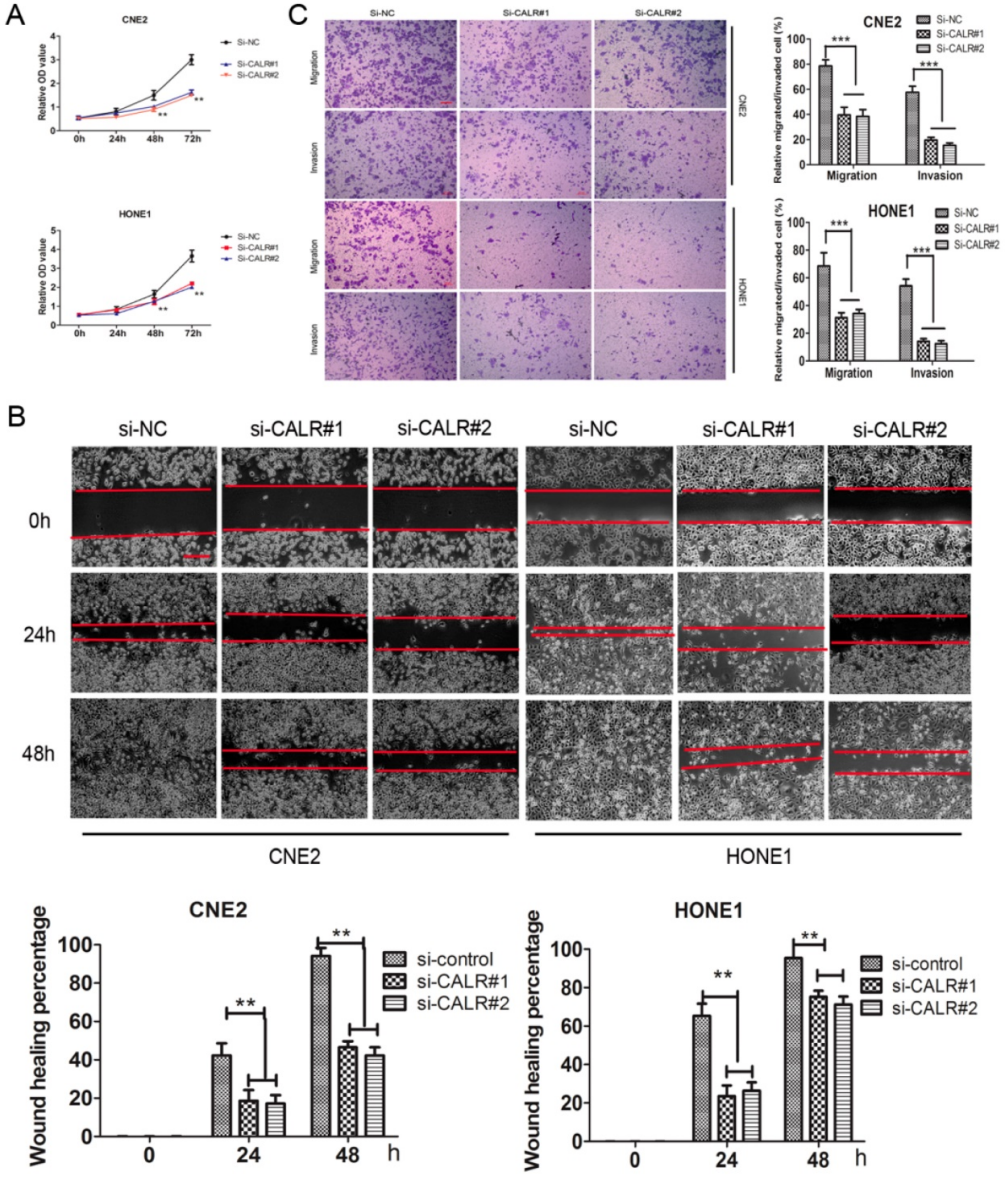
** Knockdown of CALR inhibits the cell proliferation, migration and invasion of NPC cells.** In order to silence the expression of CALR, CNE2 and HONE1 cells were transfected with CALR interfering plasmids (si-CALR#1, si-CALR#2). The proliferation of CNE2 and HONE1 cells was analyzed by Cck-8 assay (A), cell migration of CNE2 and HONE1 cells was analyzed using scratch wound assays (B), and further confirmed by transwell assays without a diluted Matrigel coating (C). Cell invasion of CNE2 and HONE1 cells was analyzed using transwell assays with a diluted Matrigel coating(C). Error Bar = SD, **P < 0.01, ***P < 0.001 compared to vector, independent Student's t-test.

We also explored the effect of CALR knockdown on NPC cell proliferation, migration and invasion. Cck8 assay showed that knockdown of CALR significantly inhibited the proliferation of CNE2 and HONE1 cells compared to the control group (Fig. 3A). The scratch wound-healing assay showed that compared with the empty vector group, knockdown of CALR in CNE2 and HONE1 cells led to slowly wound healing (Fig. 3B), and cell migration was significantly weakened (Fig. 3C). Matrigel invasion assays also showed that knockdown of CALR could significantly reduce the invasiveness of CNE2 and HONE1 cells (Fig. 3C). The results illustrated that the expression of CALR promoted CNE2 and HONE1 cell proliferation, migration and invasion.

### Knockdown of CALR suppressed Stat3 signaling pathways in CNE2 and HONE1 cells

**Figure 4:**
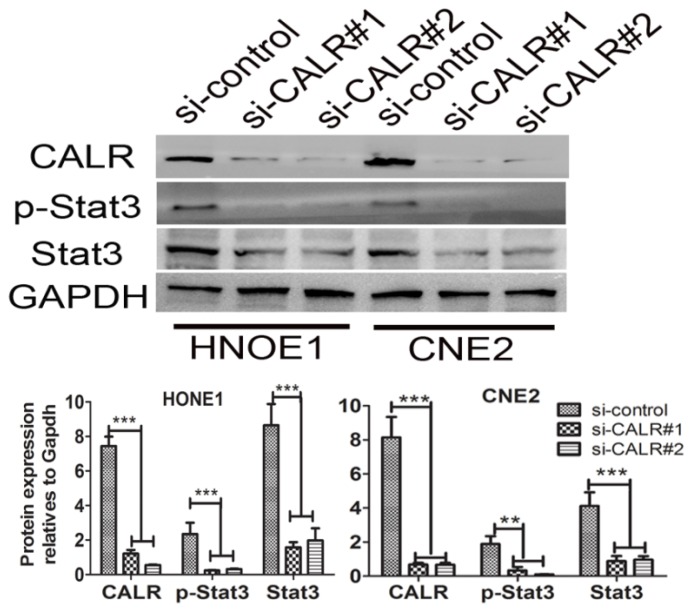
** Knockdown of CALR suppressed the expression of Stat3 in NPC cells.** The expression of CALR, Stat3 and p-Stat3 in CNE2 and HONE1 cells transfected with CALR interfering plasmids (si-CALR#1, si-CALR#2) and control vector were detected by western blotting.

Given to CALR is an upstream regulator of signal transducer and activator of transcription 3 (Stat3)^19^, we detected whether knockdown of CALR suppressed the activation of Stat3 signaling pathways in CNE2 and HONE1 cells. As illustrated in Fig. 4, the expression of Stat3 was detected by western blotting. Compared with the empty vector group, the levels of total Stat3, p-Stat3 protein expression significantly decreased in the CALR silencing CNE2 and HONE1 cells. The result indicated that CALR expression could activate Stat3 signaling pathway, thereby promoting the cell proliferation, migration and invasion of CNE2 and HONE1cells.

## Discussion

NPC is one kind of head and neck cancer that arise from cells in nasopharynx, which is typically diagnosed at advanced clinical stages, and resulting in poor outcomes^1^. Thus, it is important to explore the potential biomarkers for the diagnosis, prognosis, and treatment of NPC. In this study, CALR has been preliminarily verified as a potential molecular marker in NPC.

Our studies have indicated that LPLUNC could work as a potential tumor suppressor gene in NPC^15^, ^16^, ^18^, and we identified that CALR was one of differentially expressed genes using proteomic techniques in LPLUNC1 overexpression NPC cells, which was down-regulated by LPLUNC1, suggesting CALR plays an important role in the development of NPC and might be involved in the inhibitory roles of LPLUNC1 in NPC^14^. CALR (CRT) protein is one of the major calcium-binding protein in endoplasmic reticulum. CALR can locate in the endoplasmic reticulum, also locate in membrane and secret out of the cell. The biological function is mainly involved in the regulation of calcium balance, properly folded proteins, apoptosis, adhesion, and physiological and pathological processes of angiogenesis and autoimmune reactions^7^, ^20^. Studies have shown that CALR protein expresses in a variety of tumor cell surface, which considered as an “eat-me” signal and promotes phagocytic uptake of cancer cells by immune system, resulting in inducing immunogenic apoptosis and triggers anti-tumor immune responses^8^, ^10^, ^21^-^23^. Recently, increasing evidences also indicate that CALR has great impacts on the tumor formation and progression of various cancers, manipulation of CALR levels profoundly affects cancer cell proliferation and angiogenesis^10^, ^21^, ^22^, but the role of CALR in cancer cells remains controversial^21^. Many reports reveal that CALR protein is highly expressed in esophageal cancer^24^, pancreatic cancer^25^, ^26^, breast cancer^27^, bladder cancer^12^, ^28^ and other tumors, suggesting CALR plays a protumorigenic role. While downregulation of CALR has also been observed in endometrial cancer^29^, colon adenocarcinomas^30^, prostate cancer^31^, showing CALR has anti-tumourigenic activity. Thus, the relationship between CALR and cancer requires further investigation. Considering the current evidence, the role of CALR expression in different tumors is unclear, especially, it remains unclear whether CALR may act as a biomarker for NPC patients or not. In this study, we examined the relationship between alterations in CALR expression and the prognosis of patients with NPC. We found that the expression of CALR was significantly increased in NPC tissues, suggesting that CALR expression plays a protumorigenic role in NPC.

The expression level of CALR was apparently related with the pathological grade of the tumor, lymph node metastasis and high microvessel density in various cancer, and resulted in poor patient survival 23, 32. It means that CALR may promote tumor angiogenesis, accelerate tumor invasion and metastasis^24^. CALR overexpression enhanced angiogenesis, migration and metastasis of gastric cancer cells^13^, ^33^. High expression of CALR is highly associated with invasion and metastasis, apoptosis resistance and poor prognosis in esophageal squamous cell carcinoma (ESCC), the capabilities of ESCC movement, invasion and resistant to apoptosis was significantly decreased after blocking endogenic expression of CALR^24^, ^34^. CALR expression was also higher in the more aggressive MDA-MB-231 cells compared with MCF-7 cells at both the mRNA and protein levels^35^, ^36^, siRNA-mediated knockdown of CRT expression significantly decreased invasiveness of DADS-treated HL-60 cells^37^, ^38^. In this study, we analyzed the clinical significance of CALR expression in NPC tissues. Consistent with these reports, we found that the expression of CALR was positively correlated with the clinical stage and metastasis in NPC. Survival analysis showed over-expressed CALR in NPC patients had poor prognosis, with a shorter PFS and OS. COX multivariate regression analysis showed high expressions of CALR is important biomarkers associated with poor prognosis in NPC patients. Furthermore, we found that knockdown of CALR significantly inhibited the proliferation, migration and invasion of CNE2 and HONE1 cells. The results suggested that CALR played important roles in morbidity and progression of NPC.

Numerous studies have demonstrated that CALR is an upstream regulator of Stat3^11^, ^19^. In esophageal squamous cell carcinoma, CALR works as the upstream regulator of STAT3 promotes cell motility and enhances resistance to anoikis^34^. It is well known that Stat3 is a point of convergence for numerous oncogenic signaling pathways^39^-^41^. Stat3 is constitutively activate in both tumor cells and immune cells, and this activation promotes tumor survival and growth by increasing the capacity of tumors to evade the immune system^40^, ^42^, ^43^. Interestingly, our previous study has demonstrated that LPLUNC1 overexpression significantly inhibited Stat3 signaling pathway induced by IL-6^18^, while CALR was down-regulated by LPLUNC1^14^. Therefore, there may be a close relationship between CALR and Stat3 in NPC, but the regulatory effect of CALR on Stat3 is still unclear. In the current study, we found that knockdown of CALR could significantly suppress the activation of Stat3 signaling pathway in CNE2 and HONE1 cells, suggesting CALR could activate Stat3, CALR-Stat3 signaling pathway has been involved in the initiation and progress of NPC and the inhibitory roles of LPLUNC1 in NPC, which will be further investigated in the future.

In summary, we found that CALR was significantly up-regulated in NPC tissues, CALR high expression was positively related to advanced clinical stage, metastasis and poorer prognosis in NPC. Knockdown of CALR significantly inhibited the proliferation, migration and invasion via inactivation of Stat3 signaling pathway in CNE2 and HONE1 cells. CALR may be regarded as biomarkers of malignant progression and poor prognosis, which might become novel therapeutic targets to prevent the progression of NPC. However, the function of CALR in NPC need to be further investigated *in vivo* and *in vitro*.

## Figures and Tables

**Table 1:** Relationships between expression levels of CALR and clinicopathologic characteristics

Characteristics	Cases	CALR		
		High	Low	*P* Value
Normal	131	22	109	
Age				0.612
≤48	66	10	56	
>48	65	12	53	
Gender:				0.306
Male	95	14	81	
Female	36	8	28	
Tumor	256	164	92	
Age				0.123
≤48	125	86	39	
>48	131	78	53	
Gender:				0.325
Male	200	125	75	
Female	56	39	17	
Metastasis	164	116	48	0.003
No metastasis	92	48	44	
Stages I+II	115	65	50	0.023
Stages III+IV	141	99	42	

**Table 2:** Cox regression analyses of the various factors associated with PFS and OS in NPC patients

Variables	progression-free survival			overall survival		
	HR	95% CI	P	HR	95% CI	P
Gender (Female/Male)	0.546	0.248-1.200	0.132	0.492	0.221-1.096	0.083
Age(≤48/>48)	0.710	0.380-1.327	0.283	0.595	0.316-1.120	0.108
Stage(I+II/III+IV)	2.430	0.810-7.289	0.113	2.466	0.868-7.003	0.090
Metastasis No metastasis	0.529	0.169-1.655	0.274	0.476	0.162-1.400	0.178
CALR(High/Low)	2.564	1.217-5.405	0.013	3.179	1.464-6.902	0.003

HR, hazard ratio; 95% CI, 95% confidence interval

## Abbreviations

CALR/CRT: calreticulin
NPC: nasopharyngeal carcinoma
PFS: progression-free survival
OS: overall survival
LPLUNC1: long palate, lung, and nasal epithelium clone
NPE: non-tumor nasopharyngeal epithelium
IHC: Immunohistochemistry
Stat3: signal transducer and activator of transcription 3
